# Exercise Training Enhances Angiogenesis-Related Gene Responses in Skeletal Muscle of Patients with Chronic Heart Failure

**DOI:** 10.3390/cells10081915

**Published:** 2021-07-28

**Authors:** Andrea Tryfonos, Giorgos Tzanis, Theodore Pitsolis, Eleftherios Karatzanos, Michael Koutsilieris, Serafim Nanas, Anastassios Philippou

**Affiliations:** 1 Department of Physiology, Medical School, National and Kapodistrian University of Athens, 11527 Athens, Greece; a.tryfonos@external.euc.ac.cy (A.T.); mkoutsil@med.uoa.gr (M.K.); 2 Clinical Ergospirometry, Exercise & Rehabilitation Laboratory, Evaggelismos Hospital, National and Kapodistrian University of Athens, 10676 Athens, Greece; gs.tzanis@gmail.com (G.T.); lkaratzanos@gmail.com (E.K.); serafimnanas@med.uoa.gr (S.N.); 3 First Department of Intensive Care, Medical School, National and Kapodistrian University of Athens, 11527 Athens, Greece; theodorepitsolis@yahoo.com

**Keywords:** cardiac rehabilitation, heart failure, angiogenesis, skeletal muscle, capillarization

## Abstract

Peripheral myopathy consists of a hallmark of heart failure (HF). Exercise enhanced skeletal muscle angiogenesis, and thus, it can be further beneficial towards the HF-induced myopathy. However, there is limited evidence regarding the exercise type that elicits optimum angiogenic responses of skeletal muscle in HF patients. This study aimed to (a) compare the effects of a high-intensity-interval-training (HIIT) or combined HIIT with strength training (COM) exercise protocol on the expression of angiogenesis-related factors in skeletal muscle of HF patients, and (b) examine the potential associations between the expression of those genes and capillarization in the trained muscles. Thirteen male patients with chronic HF (age: 51 ± 13 y; BMI: 27 ± 4 kg/m^2^) were randomly assigned to a 3-month exercise program that consisted of either HIIT (*N* = 6) or COM training (*N* = 7). Vastus lateralis muscle biopsies were performed pre- and post-training. RT-PCR was used to quantify the fold changes in mRNA expression of vascular endothelial growth factor (VEGF), vascular endothelial growth factor receptor 2 (VEGFR-2), hypoxia-inducible factor 1 alpha (HIF-1α), angiopoietin 1 (Ang-1), angiopoietin 2 (Ang-2), angiopoietin receptor (Tie2), and matrix metallopeptidase 9 (MMP-9), and immunohistochemistry to assess capillarization in skeletal muscle post-training. There was an overall increase in the expression levels of VEGF, VEGFR-2, HIF-1α, Ang2, and MMP9 post-training, while these changes were not different among groups. Changes in capillary-to-fibre ratio were found to be strongly associated with Tie2 and HIF-1α expression. This was the first study demonstrating that both HIIT and combined HIIT with strength training enhanced similarly the expression profile of angiogenic factors in skeletal muscle of HF patients, possibly driving the angiogenic program in the trained muscles, although those gene expression increases were found to be only partially related with muscle capillarization.

## 1. Introduction

Whilst heart failure (HF) is fundamentally a disorder that impacts central haemodynamics [1], peripheral vascular and muscular alternations, including skeletal myopathy, have been associated with the clinical severity of HF patients [2]. Indeed, reduced exercise capacity, which is a hallmark of HF and has been associated with poor prognosis, cannot be fully explained by the decrease of left ventricular ejection fraction (LVEF) in these patients [2,3]. These observations further support the importance of muscle hypothesis in HF-related exercise intolerance [4,5].

Exercise-based cardiac rehabilitation, given its well-established benefits for the improvement of exercise capacity, has been proposed as a safe and effective therapeutic strategy in HF [6,7]. Specifically, aerobic exercise, in the form of either continuous or even high-intensity-interval-training (HIIT), has been traditionally employed in the cardiac rehabilitation programs of HF patients [6,8], while in the last decade a combined aerobic with strength training appeared to gain ground, mainly due to the benefits towards skeletal muscle atrophy. Indeed, previous work from our group showed that 3 months of combined HIIT with strength training improved exercise capacity, LVEF, and respiratory function in chronic HF patients, while it has also considerably counteracted skeletal myopathy, by increasing muscle mass, mitochondria density, and muscle strength [6]. Interestingly, we have particularly found that the capillary-to-fibre ratio in skeletal muscle had significantly increased only in HF patients who underwent the combined aerobic with strength training, whereas no changes were observed in patients that followed solely aerobic training [6].

Increased capillarization (angiogenesis) in skeletal muscle represents a crucial adaptation to regular exercise [9], as it facilitates oxygen transport in muscle [10] and thus increases exercise capacity. Previous findings in healthy individuals demonstrated increases in muscle’s vascular endothelial growth factor (VEGF), an important factor related to angiogenesis [9,11], following a short- [12] or long-term endurance training [13], whereas less is known in regards to the angiogenic adaptations to resistance exercise [14]. With respect to HF, at present only two studies have examined the angiogenic effects of exercise training on skeletal muscle, indicating an increase in mRNA and protein expression of VEGF following 8 weeks of exercise [15,16]. However, in both studies, patients performed only one-legged knee extensors training, and to the best of our knowledge, there is no study examining the impact of a traditional cardiac rehabilitation program on angiogenic adaptations of skeletal muscle in HF patients. As such, the effects of a combined aerobic and strength training program—which has been graded as a *Class I* recommendation in the treatment of HF patients according to the most recent guidelines of European Society of Cardiology-ECS (2020) [17] and American Heart Association-AHA (2017) [18]—on skeletal muscle angiogenesis remains as yet unknown.

Therefore, this study aimed to a) investigate and compare the effects of HIIT versus combined HIIT with strength training (COM) on the angiogenesis-related gene expression in skeletal muscles of stable HF patients and b) examine the potential associations between skeletal muscle capillarization and gene expression of key angiogenic factors. Given our previous findings regarding the similar skeletal muscle adaptations following HIIT or combined HIIT with strength training [6], we set the hypothesis that the expression of angiogenic factors would be increased to a similar extent in response to both exercise training protocols. In addition, we hypothesized that a positive correlation might exist between the expression of angiogenic genes and capillary-to-fibre ratio in the trained muscles of HF patients.

## 2. Materials and Methods

### 2.1. Subjects

Thirteen male patients with stable HF [age 51 ± 13 years; body mass index (BMI) 27 ± 4 kg/m^2^, LVEF 37 ± 9%], New York Heart Association (NYHA) functional class ≤III, at optimal medical treatment, consented to participate in this study. Patients were referred to the Cardiopulmonary Rehabilitation Centre from the Heart Failure Clinic of our Institution to participate in the rehabilitation program. Patients were excluded if symptom-limited cardiopulmonary exercise testing (CPET) was contraindicated according to the American Thoracic Society/American College of Chest Physicians Statement on CPET, if they had chronic obstructive pulmonary disease (COPD), severe valvular disease or peripheral vascular disease or if they had the inability to follow exercise programs due to orthopaedic problems. Informed consent was obtained from all the patients, and the study was approved by the Human Study Committee of our institution.

Some components of this study have been previously published with a special focus on the effects of exercise training on muscle hypertrophy in the HF patients [6]. The current work combines the muscle capillarization data with the expression responses of specific angiogenic genes in the trained skeletal muscles of those patients in an attempt to better understand the exercise-induced angiogenic adaptations of skeletal muscle in HF patients. Patients’ baseline characteristics and medications are given in Table 1 [6].

Values are presented as mean ± SD. ACE: Angiotensin-converting enzyme; ARB: Angiotensin receptor blockers; BMI: Body mass index; CI: Cardiac index; HF: Heart failure; ICM: Ischemic cardiomyopathy; LVEDD: Left ventricular end-diastolic diameter; LVEF: Left ventricular ejection fraction; LVESD: Left ventricular end-systolic diameter; mPAP: Mean pulmonary artery pressure; NYHA: New York Heart Association functional class; PCWP: Pulmonary capillary wedge pressure; RAP: Right atrial pressure; VO_2peak_: Peak oxygen consumption.

### 2.2. Study Design

A detailed description of the study design and method are described elsewhere [6]. Briefly, this study was a randomized clinical trial (ClinicalTrials.gov identifier: NCT02387411) and patients were randomly assigned to either the high-intensity-interval-training (HIIT, *N* = 6) group or the combined HIIT with the strength training group (COM, *N* = 7). All the participants underwent exercise training for 3 months, 3 sessions per week. Importantly, both regimes were of the same total duration (31 min). Patients in the HIIT group exercised for 3 min at 50% VO_2peak_ + 4 × (4 min at 80% VO_2peak_ + 3 min at 50% VO_2peak_), whereas in the COM group, patients exercised for 3 min at 50% VO_2peak_ + 2 × (4 min at 80% VO_2peak_ + 3 min at 50% VO_2peak_) followed by 14 min of strength training. Strength training included two exercises (leg extensions and leg curls) and each extremity was trained separately (2–4 sets, 10–12 repetitions, 30 s rest between sets, at 60–70% of 1-RM). The warm-up phase (cycling at low intensity, i.e., at 45% of VO_2peak_) and cool-down period (stretching exercises) were performed in both exercise protocols. Skeletal muscle biopsies were obtained before and after the completion of the 3-month training program from all the patients and mRNA expression of angiogenesis-related genes was examined. Changes in capillary density (capillary-to-fibre ratio) in the trained skeletal muscles of these patients were used to examine potential associations with changes in mRNA expression of angiogenic factors post-training.

### 2.3. Skeletal Muscle Biopsies

Percutaneous needle biopsies of the vastus lateralis muscle were obtained at the beginning and 48–72 h after the last exercise training session of the program, as previously described [6]. Briefly, muscle biopsies were performed at mid-thigh, under local anaesthesia (2% xylocaine), and negative suction at the time of sampling, using the Bergstrom technique [19]. Samples were snap frozen in liquid nitrogen and kept at −80°C until further analysis.

### 2.4. Capillarization

The method used for assessing capillary density has been described elsewhere [6]. Briefly, capillary density was expressed as the number of endothelial cells per muscle fibre. Endothelial cells were identified in histologic sections of skeletal muscle, by means of immunohistochemistry with the use of a specific monoclonal antibody (anti-CD31, DACO, Santa Clara, CA, USA). In this study, capillary density is expressed as the number of capillaries per muscle fibre (capillary-to-fibre ratio).

### 2.5. RNA Extraction and Semiquantitative Reverse Transcription–Polymerase Chain Reaction Analysis

RNA extraction from muscle samples and semiquantitative real-time polymerase chain reaction (RT-PCR) were used to identify differences between mRNA expression before and after the 3-monthtraining program, as previously described (6]. In brief, Trizol reagent (Invitrogen, Carlsbad, CA, USA) was used to isolate RNA from homogenized muscle samples, and the concentration and purity were determined spectrophotometrically (SpectraMax M5, Molecular Devices, LLC, San Jose, CA, USA) by absorption at 260 and 280 nm, while RNA integrity was confirmed by gel electrophoresis. Then, 1 μg of total RNA from each muscle sample was used to produce a single-stranded cDNA by means of reverse transcription (Invitrogen, Carlsbad, CA, USA). In addition, the resultant cDNA was used in RT-PCR procedures (Bio-Rad iQ5 Real-Time PCR Detection System, Hercules, CA, USA) for the determination of the mRNAs of various genes of interest, as previously described [6]. The primer set sequences used for the specific detection of each gene are presented in Table 2. To prevent detection of genomic DNA, the primer set sequences used for the specific detection of the various genes of interest were designed to lie within different exons. Specifically, primers were designed in *BLAST* against vascular endothelial growth factor (VEGF), vascular endothelial growth factor receptor 2 (VEGFR-2), hypoxia-inducible factor 1 alpha (HIF-1α), angiopoietin 1 (Ang1), angiopoietin 2 (Ang2), angiopoietin receptor (Tie2), and matrix metallopeptidase 9 (MMP9). These particular genes were selected based on previous evidence of increased mRNA expression in skeletal muscle of healthy individuals following exercise training [20,21]. Thus, in this study, we expanded the investigation of those exercise-induced angiogenic responses into HF patients who underwent traditional cardiac rehabilitation schemes such as HIIT or combined HIIT with strength training.The specificity of the primers for the corresponding gene was confirmed by the melting curve analysis of samples, where there was only one melting curve for each sample, and electrophoretic analysis of the RT-PCR products further verified the specificity of each target gene. Glyceraldehyde 3-phosphate dehydrogenase (GAPDH) was applied as housekeeping gene (internal standard) and cDNA-free reactions were used as controls for specificity for each target gene. The real-time PCR parameters were the following: Initial denaturation at 95 °C for 4 min followed by 40 cycles of 15 s at 95 °C, 30–45 s at 57−60 °C for annealing, and 30 s at 72 °C for extension.

### 2.6. Statistics

All the analyses were performed using IBM SPSS statistics for Windows, version 26.0 (IBM Corp, Armonk, NY, USA). The mRNA expression before and after the completion of the 3-month exercise training was compared via two-way repeated-measures *ANOVA* to detect differences within and between conditions. Pairwise comparisons were performed when significant main or interaction effects were detected, using Fisher’s least significant difference (LSD) test. Paired *t*-tests were used to examine potential pre-post differences within each exercise group. Associations between factors were examined using the Pearson correlation coefficient. Results are presented as mean ± SD and significance was set at *p* ≤ 0.05.

## 3. Results

### 3.1. Expression of Angiogenesis-Related Factors

**Figure 1:**
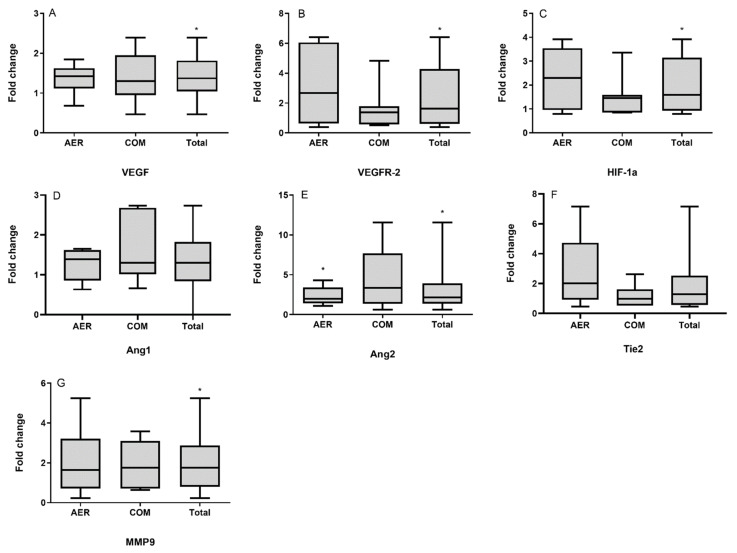
Box-plot diagrams showing the changes in the mRNA expression of vascular endothelial growth factor (VEGF; **A**), vascular endothelial growth factor receptor 2 (VEGFR-2; **B**), hypoxia-inducible factor 1 alpha (HIF-1α; **C**), angiopoietin 1 (Ang1; **D**), angiopoietin 2 (Ang2; **E**), angiopoietin receptor (Tie2; **F**), and matrix metallopeptidase 9 (MMP9; **G**) in the skeletal muscle of heart failure patients following either high-intensity-interval training (HIIT; *N* = 6) or combined HIIT with strength exercise training (COM; *N* = 7) and in the total number of patients (*N* = 13). * Significantly different compared to pre-exercise levels *p* < 0.05.

The transcriptional responses of angiogenic factors to the different exercise training protocols were examined as part of the adaptation process of skeletal muscles following those types of exercise in HF patients. It was revealed that VEGF expression was significantly increased in patients following the 3-month exercise program (*p* = 0.026), while this increase was not different between the training groups (*p* = 0.838). Similarly, there was an overall increase of the VEGFR-2 (*p* =0.028), HIF-1α (*p* = 0.011), Ang2 (*p* = 0.021), and MMP9 (*p* = 0.039) expression after the completion of the exercise program when compared to baseline levels, again without differences between the groups (*p* > 0.05). Moreover, an upward trend was observed in the overall expression of Tie2 (*p* = 0.072) and Ang1 (*p* = 0.053) following exercise training. However, these increases were not statistically different from baseline levels (*p* > 0.05). Similarly, there was no difference between the exercise groups regarding Tie2 (*p* = 0.127) and Ang1 (*p* = 0.393) expression. We further performed paired *t*-tests within HIIT and COM separately to examine whether the overall increase observed in the expression of the various angiogenic factors post-training was significant specifically within each exercise group. No pre-post differences (*p* > 0.05) were found following either HIIT or COM, except for Ang2, which was significantly increased post HIIT (*p* = 0.038). Fold changes in mRNA levels pre-to-post exercise training in HIIT group, COM group, and the total number of patients are presented in Figure 1.

### 3.2. Associations between In Vivo Capillarization and Gene Expression of Angiogenic Factors

In parallel with the effects of exercise training on the expression of angiogenesis-related factors, we also examined the potential associations between angiogenic gene expression and capillarization changes in the trained muscles. Capillary-to-fibre ratio data in both groups prior and following the 3-month HIIT or COM training program, as well as in the total number of patients are given in Table 3.

**Figure 2:**
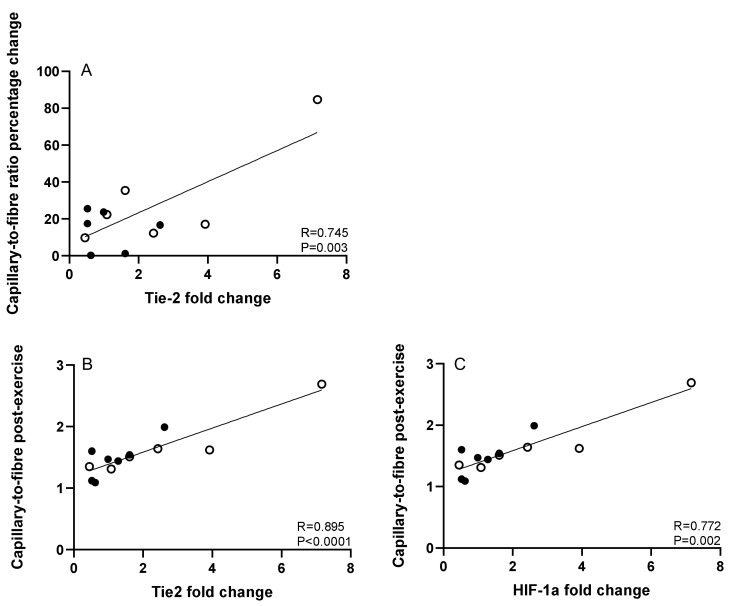
Associations between the fold change of Tie2 receptor expression (**A**) and the capillary-to-fibre ratio percentage change (%) and the absolute number of capillary-to-fibre ratio post-exercise training (**B**), as well as between the fold changes of HIF-1α expression and the absolute number of capillary-to-fibre ratio post-training (**C**) in skeletal muscles of the total number of HF patients [total *N* = 13; HIIT *N* = 6 (open cycles); and COM *N* = 7 (filled cycles)].

There was a strong correlation between the Tie2 fold change in mRNA expression post-training and the capillary-to-fibre ratio percentage change following exercise training (R = 0.745, *p* = 0.003; Figure 2).

Moreover, the absolute capillary-to-fibre ratio post-training was positively associated with HIF-1α (R = 0.772, *p* = 0.002) and Tie2 (R = 0.895, *p* <0.0001) expression fold changes. In contrast, VEGF [(a) R = 0.430, *p* = 0.142; (b) R = −0.019, *p* = 0.951], VEGFR2 [(a) R = 0.482, *p* = 0.095; (b) R = 0.338, *p* = 0.258], Ang1 [(a) R = 0.343, *p* =0.275; (b) R = 0.042, *p* = 0.892], Ang2 [(a) R = 0.124, *p* = 0.686; (b) R = −0.035, *p* = 0.909], and MMP9 [(a) R = 0.117, *p* = 0.704; (b) R = −0.147, *p* = 0.632] fold changes following exercise training were not found to correlate with either absolute capillary-to-fibre ratio (a) or capillary-to-fibre percentage change post-training (b). Lastly, several interesting associations were observed between the post-exercise training changes in the skeletal muscle expression of the angiogenic factors examined in the HF patients (Table 4).

## 4. Discussion

Muscle adaptation processes to exercise training are expected to include angiogenesis [9]. Therefore, the aim of this study was to explore and compare the angiogenic gene expression responses of a 3-month HIIT or combined HIIT with strength training exercise program in HF patients, and further examine whether these changes are associated with structural adaptations observed in the trained skeletal muscles. The main findings of our study were that both HIIT and combined HIIT with strength training resulted in similar increases in the expression of angiogenesis-related factors in skeletal muscles of HF patients. These findings indicate the activation of intramuscular angiogenic program following exercise training, and those transcriptional changes were partially correlated to changes in post-training muscle capillarization in these patients. Given that there is a growing body of evidence documenting the safety and importance of utilizing HIIT or combined HIIT with strength training in HF patients [6,22,23,24,25], our findings regarding the improved skeletal muscle angiogenesis post-training in this population are clinically important.

Specifically, we observed the upregulation of major pro-angiogenic factors, i.e., VEGF, VEGFR-2, Ang-2, and HIF-1α [11,26] in skeletal muscles of HF patients post-training, as well as several correlations between the post-exercise training changes in skeletal muscle expression of the angiogenic factors examined in these patients. Although these responses exhibited the same pattern after either HIIT or combined HIIT with strength training, increased capillarization observed only in patients who underwent the combined exercise training, suggesting that the addition of resistance exercises in a cardiac rehabilitation program may be beneficial not only for skeletal muscle hypertrophy [6], but also for improving muscle capillarization.

In particular, the increased VEGF expression and capillarization in skeletal muscles of HF patients following exercise training is in line with the findings of previous studies [15,16]. However, in those studies, the exercise-induced muscle angiogenesis was examined following 8 weeks of single-legged knee extension training. The present study is the first demonstrating the beneficial effects of 3-month typical cardiac rehabilitation schemes, such as whole-body HIIT and combined HIIT with strength training on skeletal muscle angiogenesis in HF patients.

Moreover, the current study extended the evaluation of the expression responses and their potential associations to other key regulators of angiogenesis, including VEGF receptor VEGFR-2, HIF-1α, angiopoietin system (Ang1, Ang2, their Tie2 receptor), and MMP9, in an attempt to contribute to a better understanding of the complex molecular pathways and interactions that regulate skeletal muscle angiogenesis. Specifically, we observed increased expression levels of VEGFR-2, HIF-1α, Ang2, and MMP9, but unchanged Ang1 and Tie2 mRNA levels in the trained muscles of the HF patients after 3 months of either HIIT or combined HIIT with strength training. VEGFR-2 is a crucial receptor for most of the VEGF-mediated angiogenic actions [21], particularly in exercise-induced skeletal muscle angiogenesis, as VEGFR-2 inhibition has been shown to result in abolished angiogenesis in rat skeletal muscle [27]. Higher expression of VEGFR-2 mRNA expression has been previously reported in skeletal muscle of healthy young males [20,21]. However, this is the first study showing upregulation of this receptor in skeletal muscles of HF patients, who a priori possess muscle cachexia.

In addition, skeletal muscle ischemia induced by exercise appeared to stimulate HIF-1α expression, which further promotes the expression of VEGF, reflecting a crucial role of HIF-1α in skeletal muscle capillarization [28]. Indeed, previous studies have shown an increase of HIF-1α mRNA expression levels in response to acute exercise in skeletal muscle of healthy males [29], while other studies suggested a strong positive correlation between VEGF and HIF-1α levels both at rest [30] and following acute exercise [31]. Interestingly, however, HIF-1α expression appeared not to increase in skeletal muscle in response to exercise training [32], while other studies [29] indicated that HIF-1α did not respond to acute exercise after the completion of a 4-week endurance training program in healthy males. The authors suggested that the absence of HIF-1αstimulation following regular exercise may be considered as skeletal muscle adaptation to training, given that the trained muscle is likely to no longer experience exercise-induced hypoxia [29,32]. In contrast to these findings, we found increased levels of HIF-1α expression in the trained skeletal muscles of HF patients. More interestingly, the increased expression of HIF-1α was found to be positively correlated with the absolute capillary-to-fibre ratio in the skeletal muscles of HF patients post-training. Overall, we speculate that the elevated expression levels of HIF-1α in skeletal muscles of HF patients post-training might indicate the exercise training-induced stimulation of muscle angiogenic adaptations (capillarization), possibly due to skeletal muscle ischemia that may persist during exercise in this clinical population typically characterized by peripheral myopathy [4].

With respect to the angiopoietin system, which was also explored in this study, each angiopoietin appears to have a distinct role in angiogenesis. Ang2 destabilizes endothelial cells, thus facilitating the effects of VEGF on endothelial activation, whereas Ang1 helps stabilize and maintain blood vessels [20]. Interestingly, Ang1 acts as an agonist and Ang2 as an antagonist of their common receptor, Tie2 [21]. Hence, the increased Ang2 expression along with the unchanged Ang1 and Tie2 expression levels observed in our study post-training might indicate the stimulation of endothelial cell destabilization/activation as part of the capillary remodeling and angiogenesis processes. Our findings are in line with those of previous studies following 4 weeks of cycling exercise training [20] or 5 weeks of one-legged knee extensor training [21] in healthy individuals. Moreover, and interestingly, in this study the changes in Tie2 expression levels were strongly associated with the percentage as well as the absolute changes in capillary-to-fibre ratio post-training. To the best of our knowledge, this is the first study demonstrating strong correlations between Tie2 mRNA expression levels and capillarization in human skeletal muscle, further supporting the crucial role of this angiopoietin receptor in capillary stabilization/growth. In other words, although Tie2 expression did not significantly increase following exercise training, however we assume that the close association revealed between its changes and skeletal muscle capillarization may imply the result of the competitive interactions of Ang1 and Ang2 with their receptor Tie2, i.e., capillary stability vs. growth, both of which facilitate different stages of angiogenesis. Indeed, Tie2 appears to possess two opposite roles in angiogenesis, both of which are essential in capillary stability and angiogenesis [21]. Further studies are required to draw firm conclusions regarding the exact role(s) of Tie2 in exercise training-induced angiogenesis.

Lastly, we examined the potential role of MMP9 in training-induced angiogenesis, since MMPs participate in the remodeling process of extracellular matrix (ECM) and the degradation of the basement membrane surrounding the capillary, thus facilitating the early stages of angiogenesis [20]. In particular, MMP9, as a proteolytic enzyme, plays a central role in ECM remodeling in skeletal muscle [33]. In this study, we observed an increased expression of MMP9 post-training, potentially indicating its involvement in exercise-induced adaptation and angiogenic process in skeletal muscle of the HF patients.

Overall, our findings regarding the increased transcriptional activity of pro-angiogenic factors, along with several positive correlations revealed between the expression changes of these factors post-training, suggest that exercise training, independently of its type, activates an intramuscular angiogenic program, which is functionally related to exercise-induced beneficial adaptations of the trained skeletal muscle in the patients with HF. Importantly, the similar muscle adaptations found in patients of both exercise groups, the HIIT or HIIT combined with strength training, corroborate our previous clinical findings regarding the similar improvements of exercise capacity (VO2peak, aerobic threshold, VE/VCO2 slope) in both exercise groups [6]. Thus, the impact of these exercise programs on clinical outcomes of the HF patients discussed in our previous work, e.g., their beneficial role for inducing skeletal muscle hypertrophy, could be further supported by the present findings, suggesting the therapeutic potential of both exercise interventions to confront muscle wasting and improve aerobic capacity of HF patients, as well as indicating the clinical relevance of increased skeletal muscle angiogenesis in the prognosis of HF patients.

### Limitations

The transcriptional nature of this study limits our ability to confirm the functional significance of the expression of these factors at the protein level and future studies are needed to evaluate this question.

In addition, the relatively small sample size of this study may have limited the potential to detect significant between-group differences in the expression of the factors explored. Moreover, the lack of associations between VEGF fold changes and capillary-to-fibre ratio post-training might also be explained by the small sample size of this study. Therefore, further studies are required to reach definite conclusions regarding the impact of the exercise type on skeletal muscle angiogenesis. Again, our VEGF expression data are limited only to mRNA responses, however previous studies have documented proportional changes of VEGF expression both at the mRNA and protein level in healthy individuals [20,21] and HF patients [16] following exercise training.

## 5. Conclusions

This study provided information on the exercise training-induced molecular responses, as well as the correlational relationships of angiogenesis-related factors, which are plausibly involved in a network of biological processes associated with the adaptive alterations of skeletal muscle to exercise training in HF patients. We showed an upregulation of angiogenic genes following both HIIT and combined HIIT with strength training, which, to some extent, was associated with structural adaptations, i.e., increased capillarization in the trained muscles. Given that exercise-based cardiac rehabilitation consists of a *Class I* recommendation for HF patients, more studies are needed to further characterize the molecular signature of skeletal muscle angiogenesis and adaptive remodeling following different types of exercise training in patients with HF. Increased muscle angiogenesis in these patients, who are typically characterized by muscle wasting, is crucial for enhancing their muscle health and, thus, counteracting the exercise tolerance limitations and improving their exercise capacity and prognosis.

## Figures and Tables

**Table 1:** Patients’ baseline characteristics and medications used.

	HIIT (*N* = 6)	COM (*N* = 7)
Age (years)	47 ± 13	53 ± 12
BMI (kg/m^2^)	27 ± 6	27 ± 2
HF etiology (ICM/non-ICM)	2/4	3/5
NYHA (I/II/III)	2/3/1	1/5/1
Weber class (A/B)	3/3	3/4
VO_2peak_ (mL/kg/min)	21.1 ± 4.7	20.0 ± 4.8
LVESD (mm)	43 ± 12	47 ± 13
LVEDD (mm)	59 ± 7	62 ± 9
LVEF (%)	37 ± 10	38 ± 8
PCWP (mm Hg)	9 ± 6	11 ± 8
mPAP (mm Hg)	19 ± 10	21 ± 7
RAP (mm Hg)	2 ± 2	4 ± 3
Hemoglobin (g/dL)	14.5 ± 2.4	13.9 ± 1.0
CI (L/min/m^2^)	2.2 ± 0.6	2.3 ± 0.4
**Medications (%)**		
Amiodarone	50	29
β-Blockers	100	100
Diuretics	67	71
ACE inhibitors/ARB	100	100

**Table 2:** The sequence of the specific sets of primers used in RT-PCR analyses.

Target Gene	PCR Primer Sequence	Product Size (bp)
VEGF	5′-AGGGCAGAATCATCACGAAG-3′ 5′-CACACAGGATGGCTTGAAGA-3′	163
VEGFR-2	5′-TCCCGAGTTCTGGGCATTTC-3′ 5′-GGCTCCAGTGTCATTTCCGA-3′	339
HIF-1a	5′-AAACTTGGCAACCTTGGATTGG-3′ 5′-TCCGTCCCTCAACCTCTCAG-3′	189
Ang-1	5′-ACCGGATTCAACATGGGCAA-3′ 5′-CATGGTAGCCGTGTGGTTCT-3′	281
Ang-2	5′-GACGGCTGTGATGATAGAAATAGG-3′ 5′-GACTGTAGTTGGATGATGTGCTTG-3′	264
Tie-2	5′-TGCGAGATGGATAGGGCTTG-3′ 5′-CAGAGGCAATGCAGGTGAGA-3′	440
MMP-9	5′-CAGGGAATGAGTACTGGGTCTATT-3′ 5′-ACTCCAGTTAAAGGCAGCATCTAC-3′	76
GAPDH	5′-CATCACTGCCACCCAGAAGA-3′ 5′-TCCACCACCCTGTTGCTGTA-3′	438

**Table 3:** Capillary-to-fibre ratio prior (Pre) and after (Post) high-intensity-interval training (HIIT) or combined HIIT with strength training (COM) program, and in the total number of patients. Values are presented as mean ± SD, and percentage change (%Change) from baseline. * Significantly different compared to Pre values (*p* < 0.05).

	HIIT (*N* = 6)	COM (*N* = 7)	Total (*N* = 13)
Pre	1.29 ± 0.17	1.26 ± 0.26	1.27 ± 0.21
Post	1.69 ± 0.51	1.46 ± 0.30 *	1.57 ± 0.41
% Change	30.3 ± 11.1	16.0 ± 11.1	22.6 ± 21.2

**Table 4:** Associations between the fold changes of angiogenic factors expression in the trained muscles of the patients with HF. Pearson correlation coefficient (R) and *p*-values are reported. Significant correlations (*p* < 0.05) are highlighted in bold.

	VEGF	VEGFR-2	HIF-1a	Ang1	Ang2	Tie2	MMP9
**VEGF**		R = 0.397 *p* = 0.180	R = 0.470 *p* = 0.105	**R = 0.578** ***p* = 0.049**	R = 0.374 *p* = 0.208	R = 0.347 *p* = 0.245	**R = 0.585** ***p* = 0.036**
**VEGFR-2**	R = 0.397 *p* = 0.180		R = 0.453 *p* = 0.120	R = 0.300 *p* = 0.343	R = 0.168 *p* = 0.583	**R = 0.567** ***p* = 0.043**	**R = 0.588** ***p* = 0.034**
**HIF-1a**	R = 0.470 *p* = 0.105	R = 0.453 *p* = 0.120		R = 0.515 *p* = 0.087	R = 0.259 *p* = 0.394	**R = 0.836** ***p* < 0.0001**	R = 0.358 *p* = 0.230
**Ang1**	**R = 0.578** ***p* = 0.049**	R = 0.300 *p* = 0.343	R = 0.515 *p* = 0.087		**R = 0.818** ***p* = 0.001**	R = 0.291 *p* = 0.359	**R = 0.738** ***p* = 0.006**
**Ang2**	R = 0.374 *p* = 0.208	R = 0.300 *p* = 0.343	R = 0.515 *p* = 0.087	**R = 0.818** ***p* = 0.001**		R = 0.061 *p* = 0.843	**R = 0.569** ***p* = 0.042**
**Tie2**	R = 0.347 *p* = 0.245	**R = 0.567** ***p* = 0.043**	**R = 0.836** ***p* < 0.0001**	R = 0.291 *p* = 0.359	R = 0.061 *p* = 0.843		R = 0.261 *p* = 0.390
**MMP9**	**R = 0.585** ***p* = 0.036**	**R = 0.588** ***p* = 0.034**	R = 0.358 *p* = 0.230	**R = 0.738** ***p* = 0.006**	**R = 0.569** ***p* = 0.042**	R = 0.261 *p* = 0.390	

VEGF: Vascular endothelial growth factor; VEGFR-2: Vascular endothelial growth factor receptor 2; HIF-1α: Hypoxia-inducible factor 1 alpha; Ang1: Angiopoietin 1; Ang2: Angiopoietin 2; Tie2: Angiopoietin receptor 2; MMP9: Matrix metallopeptidase 9.

## Data Availability

The data presented in this study are available on request from the corresponding author.
